# Physiological Tradeoffs of Immune Response Differs by Infection Type in *Pieris napi*

**DOI:** 10.3389/fphys.2020.576797

**Published:** 2021-01-13

**Authors:** Naomi L. P. Keehnen, Lucie Kučerová, Sören Nylin, Ulrich Theopold, Christopher W. Wheat

**Affiliations:** ^1^Department of Zoology, Stockholm University, Stockholm, Sweden; ^2^Department of Molecular Biosciences, The Wenner-Gren Institute, Stockholm University, Stockholm, Sweden; ^3^Biology Centre, Czech Academy of Sciences, Institute of Entomology, České Budějovice, Czechia

**Keywords:** infection, life history, transcriptomics, trade-offs, lepidoptera

## Abstract

Understanding the tradeoffs that result from successful infection responses is central to understanding how life histories evolve. Gaining such insights, however, can be challenging, as they may be pathogen specific and confounded with experimental design. Here, we investigated whether infection from gram positive or negative bacteria results in different physiological tradeoffs, and whether these infections impact life history later in life (post-diapause development), in the butterfly *Pieris napi*. During the first 24 h after infection (3, 6, 12, and 24 h), after removing effects due to injection, larvae infected with *Micrococcus luteus* showed a strong suppression of all non-immunity related processes while several types of immune responses were upregulated. In contrast, this tradeoff between homeostasis and immune response was much less pronounced in *Escherichia coli* infections. These differences were also visible long after infection, via weight loss and slower development, as well as an increased mortality at higher infection levels during later stages of development. Individuals infected with *M. luteus*, compared to *E. coli*, had a higher mortality rate, and a lower pupal weight, developmental rate and adult weight. Further, males exhibited a more negative impact of infection than females. Thus, immune responses come at a cost even when the initial infection has been overcome, and these costs are likely to affect later life history parameters with fitness consequences.

## Introduction

Pathogens and parasites exert strong selection pressures upon hosts for their survival. Mounting an immune response is energetically costly in terms of physiology, development, and reproduction, and due to organism being limited by finite resources, often resulting in trade-offs between immune response and the other life history traits (Sheldon and Verhulst, 1996; Ahmed et al., 2002; Zuk and Stoehr, 2002; Freitak et al., 2003; Ardia et al., 2012). An energetically costly life-history trait among animals is insect metamorphosis, wherein the organism experiences a dramatic shift in their overall morphology, physiology, and often environment (e.g., from terrestrial larvae, to airborne butterfly; Russell and Dunn, 1996). For insect species living in temperate zones, metamorphosis is often directly followed by a developmental arrest called diapause, for which the insect needed to have acquired sufficient energy reserves before the onset of winter (Hahn and Denlinger, 2007). Despite immunity, metamorphosis and diapause being essential and energetically costly life-history traits, the impact of infection before metamorphosis and diapause upon life-history traits later in life has rarely been studied. Although reallocating resources to fighting pathogens is expected to incur costs, to what extent variation in the type of infection mediates such tradeoffs between immune response and later life history allocation is under explored.

Insects occur in a wide variety of environments, where they are exposed to physical trauma resulting in wounds, frequent attack by parasites and pathogens, and sometimes both. The insect immune response is divided into humoral and cellular defense responses. Humoral defenses consist of the production of antimicrobial peptides via Toll and IMD signaling pathways, and enzymes like phenoloxidases (producing melanin) and reactive intermediates products (Lemaitre and Hoffmann, 2007). The cellular defenses of insects are hemocyte-mediated responses, such as phagocytosis and encapsulation (Strand, 2008). While an immune response can be effective, such a response shifts metabolic priorities within the host away from regular physiological processes, to focus on immunity and wound repair (Lochmiller and Deerenberg, 2000; Dolezal et al., 2019). As a result, the cost of immunity is not only the direct metabolic cost, but also the cost of resource allocation trade-offs (Zuk and Stoehr, 2002; Adamo et al., 2008; Ardia et al., 2011, 2012). For example, developmental time, reproductive success, pupal weight, adult lifespan, all have been identified previously to trade-off with immune response, within a life stage (Boots and Begon, 1993; Thomas and Rudolf, 2010; Kingsolver and Diamond, 2011; Bajgar et al., 2015).

Any negative impact on life-history traits creates the possibility of long-term costs of successfully fighting off an infection. The complex life cycle of an insect could be delayed or disrupted, and ultimately negatively affect its lifetime survival and reproductive success. In order to fully understand the costs of the immune response, and its potential cost to other component of the organism’s life history, an integration between phenotypic and physiological analyses is needed across these complex life stages (Coustau et al., 2000; Zuk and Stoehr, 2002).

The immune response competes for resources with other energy consuming processes, like metamorphosis and diapause. Metamorphosis is a critical period in which energy stores established from larval feeding are allocated between fueling pupal development and supporting the needs of the adult for reproduction and survival (Boggs and Freeman, 2005; Boggs, 2009; Merkey et al., 2011). Despite a sharp decline in metabolic rate when entering metamorphosis, metamorphosis is a costly process, for example, in *D. melanogaster* pupae consumed 35 and 27% of their lipid and carbohydrate reserves (Merkey et al., 2011). Despite being described as a developmental arrest, diapausing individuals are not running slower than non-diapausing insects, they are following an alternative development pathway with its own unique metabolic demands (Koštál, 2006; Hahn and Denlinger, 2007).

Insects that have not accumulated enough reserves to survive diapause either (i) die during diapause or post-diapause development, (ii) postpone diapause and try to produce one more generation, or (iii) terminate diapause early when the energy reserves are low (Hahn and Denlinger, 2007). To our knowledge, it is currently unknown whether negative effects of infection and wounding during the critical larval stage influences metamorphosis and diapause, although this is expected. The majority of the immune eco-physiological studies in insects are done on a phenotypic level, measuring either the immune response, or life history characteristics. No studies have looked at the initial immune response on a molecular level, to see if gene expression patterns during this phase could explain variation in life history data observed later in life. Transcriptome analysis can provide physiological insights into biological processes that are active in tissues, wherein a change in the expression pattern of a gene is an indication of molecular functions that are changing over time. In the case of infection studies, such insights could reveal indications of trade-offs in functional pathways. In sum, few studies have tried to integrate physiological insights via RNA-Seq with phenotypic measures of life history, across infection titers of different infection types.

Here, to gain insights into the long-term consequences of infection during critical phases of development, we immunologically challenged lepidopteran larvae preparing to pupate for diapause, after which we measured key life history traits, as well as looked at their initial transcriptomic profile of their immune response. To gain a more general understanding, we used two different types of immune challenges that are commonly found in the literature, live gram-negative (*Escherichia coli*) and gram-positive (*Micrococcus luteus*) bacteria, as gram-positive and gram-negative bacteria elicit different patterns of immunoregulatory activity. In addition, we included injection with PBS as a control for wounding, against which we could then look for the additional impacts of bacterial infection. In our RNA-Seq experiment, we specifically remove such wounding effects from our analyses.

## Materials and Methods

### Study Organism and Experimental Design

The green veined white butterfly (*Pieris napi*) is a widespread generalist butterfly. It occurs throughout Europe and in the temperate zone of Asia (GBIF, 2017). For this study, female butterflies were collected in northern Sweden (Abisko township) and Southern Sweden (Kullaberg park, Skåne) in August 2014. These females were transferred to Stockholm University where they were allowed to lay eggs on Garlic mustard (*Alliaria petiolata*). Larvae from wild-caught females were fed on *A. petiolata* leaves until pupation in a climate-controlled room (Light:Dark 12:12 h, 17°C). Pupated offspring were placed in cold conditions (4°C) 21 days after pupation.

In order to test the effects of infection with gram-positive or gram-negative bacteria on survival, pupae from Abisko were taken out of diapause in April 2014 and placed in a climate-controlled room (L:D 23:1, 23°C). Unrelated males and females each received a unique identifier before release into the mating cage, and were fed ad lib on 20% sugar solution. Adults were observed every hour to ensure parentage. Once mated, females were placed in individual cups with *A. petiolata* for oviposition. The leaves were exchanged twice a day, until females stopped laying eggs. The offspring from four females that produced the highest number of eggs were chosen for the experiment. The eggs were kept in containers and placed in climate chambers to develop, and grown under diapausing conditions (L:D 8:16, 17°C). After reaching third instar, larvae were moved to individual cups containing *A. petiolata* and checked daily to monitor development.

Once larvae reached the second day of 5th instar they were sexed and randomly divided among 8 treatment groups (Supplementary Figure 1). One treatment was injected with 10 μl of sterilized phosphate-buffered saline (PBS), to act as a trauma control. To investigate the effect of different doses of bacteria; three treatment-groups were injected with the live gram-negative *E. coli* (10^4^, 10^5^, and 10^6^), another three treatment-groups were injected with the gram-positive *M. luteus* (10^4^, 10^5^, and 10^6^), and the final treatment-group was left as uninjected controls. Details described below. Larvae were weighed to the nearest 0.1 mg and afterward anesthetized by chilling them in containers on ice for 5 min prior to injection. The syringe needle (Hamilton SYR 10 μL 701 ASN) was sterilized by rinsing three times each in two tubes of 95% ethanol, followed by one tube of sterile H_2_O. The injection was done at an angle less then 45° behind the hind abdominal proleg, which was sterilized with a 95% ethanol swab beforehand (Hussa and Goodrich-Blair, 2012). Control individuals were weighed and anesthetized without injection. Larval survival was monitored twice daily, until all surviving individuals reached pupation.

For the RNA-seq experiment, larvae from Skåne, southern Sweden (Kullaberg; 56°18′N, 12°27′E 10^9^), from the same stock as Lehmann et al., 2017, were taken out of diapause and reared in the identical conditions as above. The injection treatment was identical as above, the only deviation being the treatments, instead of eight, there were only three treatment groups: PBS, *E. coli* 10^6^, *M. luteus* 10^6^. Larva were sampled at 3, 6, 12, and, 24 h after injection. Individuals were sampled by placing them in a 1.5 mL tube, and submerged into liquid nitrogen after which they were stored in −80°C.

### Live Bacteria

For both experiments, live *E. coli* DH5 alpha (1 OD = 8.3E + 08 CFU/ml) and *M. luteus* CCM 169 (1 OD = 1E + 07 CFU/ML) were obtained from stock. The optical density (OD) was determined for both bacteria. On a daily basis, an inoculating loop was used to transfer a single colony from the LA plate to 3 ml LB broth. The culture was grown overnight at 37°C with shaking at 250 rpm. A serial dilution was then performed to determine the number of colony forming units (CFUs) and optical density of the stock bacteria. The optical density of the broth was quantified in the spectrophotometer and used to dilute the samples to 10^4^, 10^5^, and 10^6^. The bacterial cultures were spun at 1,500 rpm for 2 min, the supernatant was discarded and the resulting pellet resuspended using 1x PBS to obtain the three doses for each bacterium.

### Life History Traits

For the life history experiment: survival, larva weight at second day of 5th instar, time to develop to pupa after treatment, pupal mass 23 days after pupation, pupal mass 247 days after pupation, time to eclose after diapause, adult whole-body weight, abdomen weight and thorax weight were recorded (Supplementary Figure 1). All pupae were exactly 224 days in the cold treatment, after which they were weighed to the nearest 0.1 mg and placed in a climate-controlled room (L:D 23:1 h photo cycle, 23°C). Pupae were checked twice a day to obtain accurate eclosion date. After eclosion the adults were put into 4°C for 1 day so that they could drop their meconium, after which they were weighed to obtain adult whole body, thorax and abdomen mass. Individuals were sexed in all life stages.

### Statistical Analysis

Statistical analyses were performed in JMP 14 (SAS) (2018). For all analyses, data were checked for normality and heteroscedasticity where applicable. For each regression analysis (GLM), all variables were entered into the model, and non-significant variables were eliminated in a stepwise manner until the model contained only significant variables, or there was no change in the fit of the model (Akaike information criterion). Developmental rates, weights, and body ratios were investigated using Kruskal–Wallis each pair comparisons. For weight data, previous studies have revealed a strong sex difference in butterflies, therefore all weight data was analyzed separately for each sex. Family was tracked for each individual larva, and added to each initial statistical test as variable, but as there was no significant effect found, these are not reported in the final analyses.

### RNA Isolation and Sequencing

Total RNA was extracted from a total of 72 larvae, six per time point, per treatment. RNA was purified with the Direct-zol RNA MiniPrep (Zymo, Irvine, CA, United States) as per manufacturer’s instructions. Quality and quantity of the total RNA purified were determined using Experion equipment (Bio-Rad, Hercules, CA, United States) and a Qubit instrument (Thermo Fisher Scientific, Waltham, MA, United States) Due to technical error one individual of the PBS treatment failed, therefore this sampling point only has five replicates, which resulted in the final total of 71 individuals sequenced. Library preparation, sequencing and data processing of the RNA was performed at the National Genomics Infrastructure Sweden (NGI Stockholm) using strand specific Illumina TruSeq RNA libraries with poly-A selection (Illumina HiSeq HO mode v4, paired-end 2 × 125 bp).

### Transcription-Level Expression Analysis

BBduk v37.31^1^ was used to trim adapter sequences and filter to a base pair quality score of 20. Transcript-level expression analysis was done following protocol provided by Pertea et al., 2016. Briefly, reads were mapped using HISAT2 v2.1.0 (Kim et al., 2015) to the *Pieris napi* genome v1.1 (Hill et al., 2019). Samtools sort v1.7 was used to sort the file, after which it was transformed into a BAM file (Li et al., 2009). Transcripts were assembled using StringTie v1.3.4 (Pertea et al., 2016), and the *Pieris napi* v1.1 annotation file in GTF format. This resulted in an updated GTF annotation file for the *P. napi* genome. Transcript abundances were estimated for each sample using StringTie v1.3.4, and the merged transcript file as input. A gene-level read count matrix was generated using the prepDE.py script provided as part of the StringTie package, using an average read length of 125 https://ccb.jhu.edu/software/stringtie/dl/prepDE.py. Sample relationships were examined using PtR as part of Trinity v2.8.3 (Grabherr et al., 2011; Haas et al., 2013). For differential expression analysis, pairwise comparisons between all samples were conducted using DESeq2 at the gene level, including VST transformation (Love et al., 2014). Two type of DE analysis were performed, in the first analysis, genes were determined to be significantly differentially expressed when having an adjusted at a log fold change (FC) of 0, and a *p*-value of 0.001 or lower, representing a false discovery rate (FDR) of 0.1% on a *p*-value of 0.001. In the second analysis genes were determined to be significantly differentially expressed when having an adjusted at a log fold change (FC) of 2, and a *p*-value of 0.001 or lower.

### Cluster Analysis

Two type of clustering of expression profiles over time were performed. First, to identify the overall transcription profile of the first 24 h after infection and injury in a larva, a time series analysis was conducted on genes that were differentially expressed (DEGs, (logFC 0; FDR < 0.001) between 3, 6, 12, and 24 h after injection within each treatment (*PBS*, *E. coli*, or *M. luteus*).

Secondly, to exclude the genes being up/downregulated as a result of the injection of PBS, and to specifically identify the genes involved with the immune response, we compared the bacterial treatment with their PBS counterparts for each time point (Supplementary Table 1). Subsequently, to investigate the expression dynamics related to the immune response over 24 h after treatment, a time series expression cluster analysis was conducted by the bacterial treatment in comparison with the PBS treatment (logFC > 2 and logFC < -2; FDR < 0.001).

For both cluster analyses, the R package Mfuzz was used to perform the clustering using the Fuzzy c-means method (Futschik and Carlisle, 2005). First, the number of clusters were determined using K-means and the within cluster sum of squared error (SSE; elbow method) in each data set. Briefly, this method determines the sum of the squared distance between each member of a cluster and its cluster centroid, and at a certain number of clusters number the SSE will not significantly decrease with each new addition of a cluster, which provides the suitable number of clusters. Fuzzy c-means assigns each data-point a cluster membership score, where being closer to the cluster center means a higher score, and these scores are used to position the centroids. This results in a robust clustering, since low scoring data points have a reduced impact on the position of the cluster center, and as a result noise and outliers have less influence. After which the centroids were correlated to ensure that the clusters separated properly, with no correlation score above 0.85.

### Gene Set Enrichment

Gene set enrichment analysis (GSEA) was performed on the time series cluster analysis with the topGO v2.24.0 R package (Alexa et al., 2006). Genes were classified as belonging to a cluster when having a cluster score of >0.6, indicating that of all clusters, the gene belongs most to that particular cluster. The genes considered in the GSEA were those with existing GO annotations in the annotation of the genome assembly (Hill et al., 2019). In topGO, the nodeSize parameter was set to 5 to remove GO terms having fewer than five annotated genes, and other parameters were run on default. GSEA were performed using the parentchild algorithm, which takes the current parents’ terms into account. Furthermore, the ontology level was run for both biological processes and molecular function. Data were summarized and visualized using Revigo and Treemap in R (Supek et al., 2011).

For the immunity time-series analysis, the DE genes were all novel transcripts constructed by StringTie, and therefore had no GO annotation in our *P. napi* v1.1 genome. Also, compared to the previous analysis, this analysis contained fewer genes. Therefore, instead of a traditional GSEA using GO terms, the genes were manually annotated. First, the exonic regions were extracted from the genome using GFFread (obtained from the Cufflinks suite at http://cole-trapnell-lab.github.io/cufflinks/; Trapnell et al., 2010). These exonic regions were searched against the Uniprot protein database (Suzek et al., 2007), using blastx (thresholds: single hit, bitscore >60 and *E*-value < 0.0001; Altschul et al., 1990).

## Results

We first investigated the longer life history consequences of infection, and the effect of infection on survivorship and Darwinian fitness proxies (e.g., weight gain, developmental rates) throughout developmental stages. Then we investigated the late larval instar response to infection, using RNA-seq to characterize the immune system induction and assess whether there was evidence for physiological trade-offs.

### Life History: Survival and Timing of Death

**FIGURE 1:**
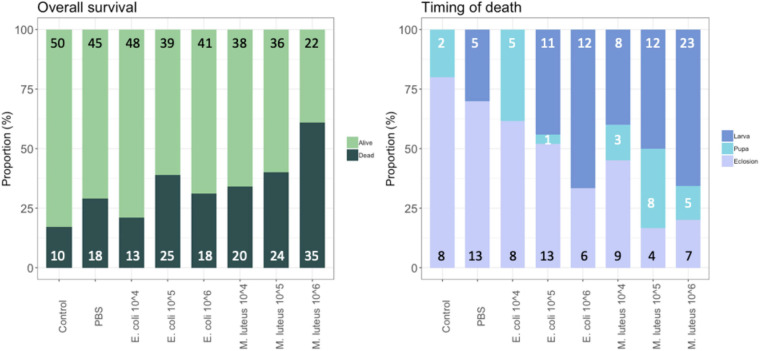
Proportion of individuals surviving and dying per treatment, with the total number per class added in the graph **(left)**. For the individuals that died, timing of death was classified as either occurring during infection (death in the larval stage), during diapause (death as a pupae), or during eclosion **(right)**. Graph shows the proportion of each of these groups per treatment, with the total numbers given in the graph.

To determine the effect of live bacteria on survivorship and Darwinian fitness proxies (e.g., weight gain, developmental rates), 5th instar larvae were injected with either PBS as a control, the gram-negative *E. coli*, or the gram-positive *M. luteus*, as in the previous section. However, here we explored the effect of injection in a dose responsive manner, using concentrations of live bacteria from 10^4^ to 10^6^. First, in order to evaluate whether the injection itself had an effect, mortality in the PBS treatment was compared to mortality in the control treatment. Although not significant, the overall mortality was 15% higher for the group injected with PBS, compared to the non-injected control individuals (*X*^2^ = 3.46, *N* = 125, df = 1, *P* = 0.06). Next, bacterial treatment showed a significant effect on overall survival (GLM with groups (control, PBS, bacterial injections): *X*^2^ = 49.46, df = 10, *P* < 0.001, Figure 1, left panel). On average *M. luteus* elicited a higher mortality than *E. coli*, and mortality increased with an increasing dose of both pathogens (Treatment: *X*^2^ = 34.87, df = 7, *P* < 0.001; Figure 1). For the individuals that died, timing of death was classified as either occurring during infection (death in the larval stage), during diapause (death as a pupae), or during eclosion. Overall, a higher dose of bacteria affected a more immediate death after infection, instead of mortality occurring at a later life-stage (Figure 1; *X*^2^ = 56.47, *N* = 165, df = 12, *P* < 0.0001).

### Life History: Developmental Rate

**FIGURE 2:**
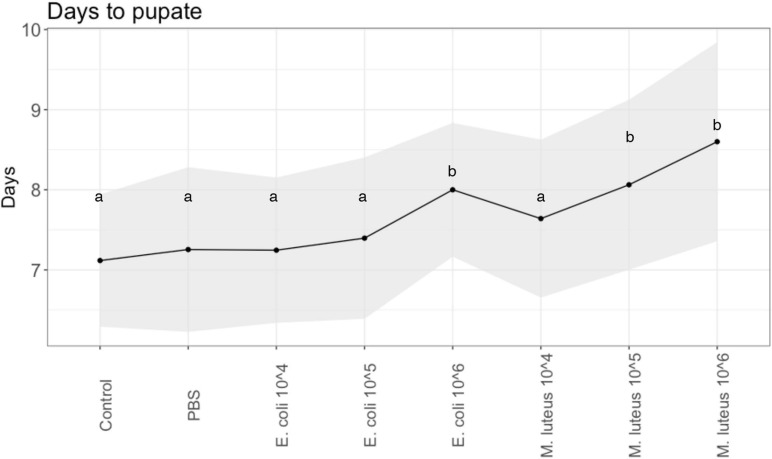
Developmental rate across injection treatments of *P. napi.* Black dots are the average per treatment, shaded areas show the standard deviations from the mean. Values not connected by the same letter are significantly different.

For the individuals that remained alive after injections, the developmental rate, i.e., the duration of time for the larvae to pupate, was significantly different between the control and the treatments (Least Squares: F-Ratio = 8.93, df = 7, *P* < 0.0001, sex = n.s.). Specifically, compared to Control (*M* = 7.1, SD = 0.81) and PBS (*M* = 7.13, SD = 0.92), the infection treatments of *E. coli* 10^6^ (*M* = 7.95, SD = 0.84), as well as with *M. luteus*, 10^5^ (*M* = 8.03, SD = 1.12) and *M. luteus* 10^6^ (*M* = 8.45, SD = 1.37) took significantly longer to turn into pupae (Figure 2), with an average increase of up to more than a day (>18%).

### Life History: Body Weights

**FIGURE 3:**
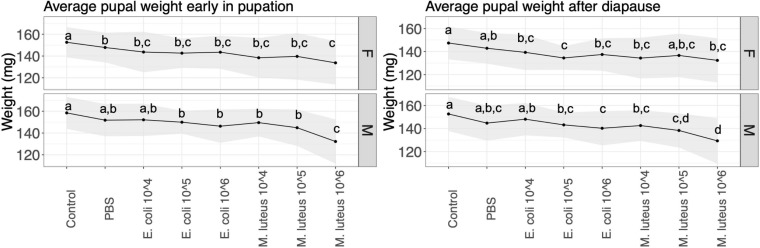
Average pupal weight over two time points per treatment. Lines show the standard deviation. **Left panel** shows the weight 23 days after pupation (the day they go into cold treatment), and the **right panel** shows the average pupa weight 247 days after pupation (stop of the cold treatment). Values not connected by the same letter are significantly different.

Pupal weight was measured at two time points, first when the pupa was 23 days old (before cold treatment), and finally when the pupa was 247 days old (when they were taken out of their cold treatment to end diapause). At 23 days there was a significant difference between control individuals and most other treatments, in both males and females (Figure 3 and Supplementary Table 2). Most notably, a 13% weight loss was observed between individuals injected with PBS and individuals injected with *M. luteus* 10^6^. The pupal weight after diapause showed a similar pattern as above, with significant differences between controls and most other treatments in both sexes, as well as a 10% weight loss difference between PBS and *M. luteus* 10^6^ (Figure 3 and Supplementary Table 2).

**FIGURE 4:**
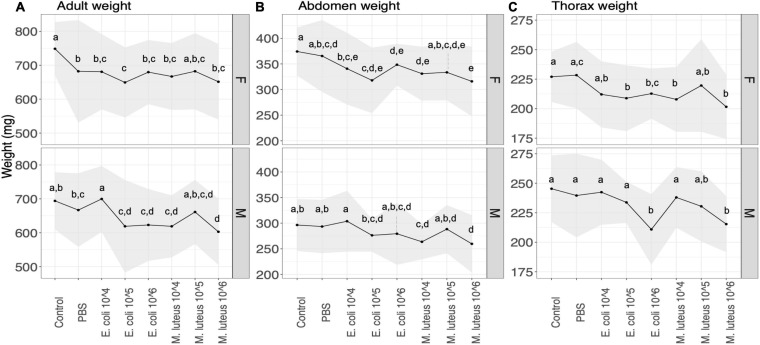
The effect of the treatment on the **(A)** weight and **(B)** abdomen **(C)** thorax weight of adult butterflies. Upper panel are females (F) lower panel are males (M). The upper panels are female (F) the lower panels males (M). The shaded area denotes standard deviations. Values not connected by the same letter are significantly different.

Adult weight after eclosion showed a significant difference between treatments in both sexes (Figure 4 and Supplementary Table 3). For females, all the treatments showed a significant decrease in weight compared to the controls, with the exception of *M. luteus* 10^5^, as well as a significant difference between PBS and *E. coli* 10^5^ (Figure 4 and Supplementary Table 3). For males, the differences were significant between the control and the infection treatments *E. coli* 10^5^, *E. coli* 10^6^, *M. luteus* 10^4^, and *M. luteus* 10^6^, as well as additional differences between *E. coli* 10^4^ and several other infection treatments (Figure 4 and Supplementary Table 3). Notably, a difference of 17% was present between PBS and *M. luteus* 10^6^ (Figure 4).

The weight of the adult abdomen was also affected by the treatment, with female abdomen being significantly lighter in the infection treatments, compared to controls, as well as between PBS, and *E. coli* 10^5^ or *M. luteus* 10^6^ (Figure 4 and Supplementary Table 3). In males this was only true for individuals treated with *M. luteus* 10^4^, 10^6^, which differed both from controls and PBS (Figure 4 and Supplementary Table 3). Thorax weight was significantly lower in females between controls and several infection treatments, as well as between PBS and a number of infection treatments (Figure 4 and Supplementary Table 3). Males showed similar patterns (Figure 4 and Supplementary Table 3). In males treated with the highest dose of either bacteria (*E. coli* 10^6^ and *M. luteus* 10^6^) had up to 12% lower thorax weight (Supplementary Table 3).

### Transcriptome Analysis of Initial Infection

The RNA-seq analysis covered across 4 time points during the first 24 h post injection (3, 6, 12, and 24 h) of 5th instar larvae, injected with either PBS as a control, the gram-negative *E. coli*, or the gram-positive *M. luteus* using a high-level of infection dose (10^6^). This dose corresponds to the highest level in our life history investigation.

**FIGURE 5:**
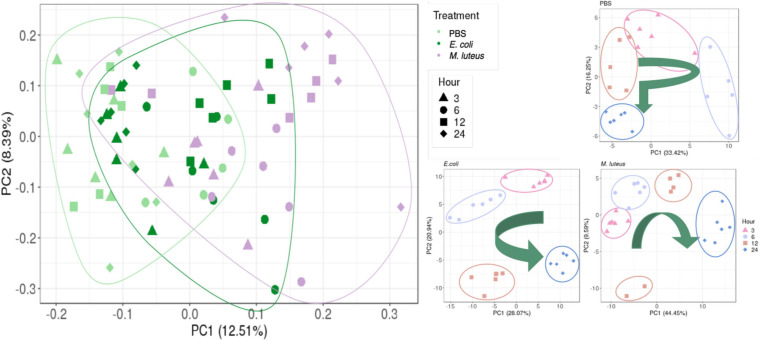
Relationship between the samples. **Left graph** shows the relationship of all three treatments. On the **right** are the comparisons of gene expression profiles when injected with PBS, *E. coli* or *M. luteus* across time. The arrows indicate the overall time progression.

First, sample relationships were assessed using a principle component analysis (PCA). The first two PCs grouped individuals within time points per treatment, which together accounted for 49–54% of the sample variance (Figure 5). The PBS samples after 12 h appear similar in their transcription as the 3 h samples. The *E. coli* samples appear to return to the initial 3 h expression profile after 24 h, whereas the *M. luteus* are on a linear trajectory along PC1, with two individuals at 12 h diverging.

### Expression Dynamics Over Time

**FIGURE 6:**
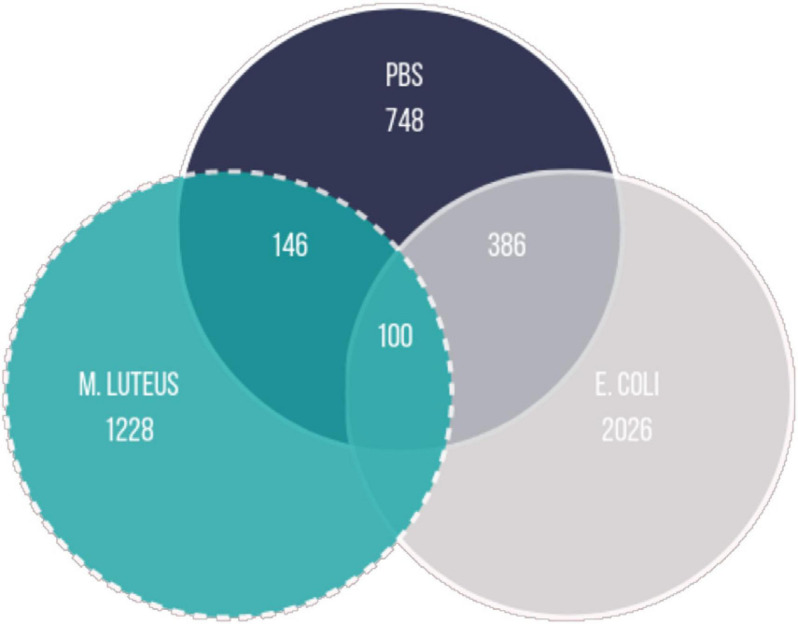
Overlap between the genes being differentially expressed in the different treatments (logFC = 0; FDR < 0.001; FC, fold change; FDR, false discovery rate).

To investigate the expression dynamics over 24 h after treatment, we performed a cluster analysis on the genes that were differentially expressed between any time points within each treatment. First, we determined the total number of genes differentially expressed between any time point in the experiment within each treatment (FDR < 0.001). The vast majority of genes showing changes in expression over time were unique to each treatment, with only a small subset of genes shared by all (*N* = 100; Figure 6).

**FIGURE 7:**
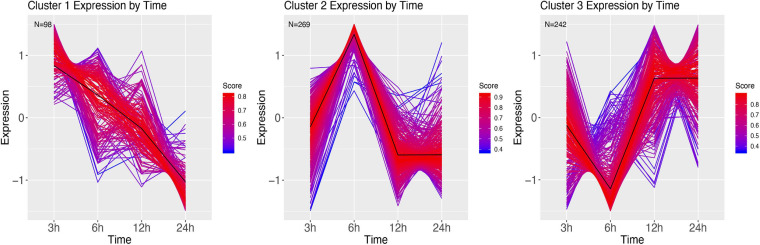
DEG clusters of larvae injected with PBS over 24 h. Color indicates cluster membership score, ranging from 0 (blue) to 1 (red). Numbers in each graph represent the number of genes in this cluster with a membership value higher than 0.6.

We next grouped the differentially expressed genes in clusters using a soft clustering approach, based on the change in their expression profile over time. Cluster estimation analysis of the PBS treatment grouped the expression patterns in three clusters (Figure 7). Cluster one shows a continuous decline in expression of genes at 3 h. GSEA revealed these genes to be involved with purine containing compound metabolism and hydrogen transport (Supplementary Figure 2). Cluster two shows higher expression at 6 h, and a return to lower expression in the next time points. GSEA revealed genes involved with the regulation of biological process and regulation, phosphorus metabolism, and cell death (Supplementary Figure 3). Finally, cluster 3 mirrors cluster 2, with a low expression at 6 h, after which expression increases for 12 h. GSEA revealed genes involved in establishment of protein localization, ncRNA metabolism and protein folding (Supplementary Figure 4).

**FIGURE 8:**
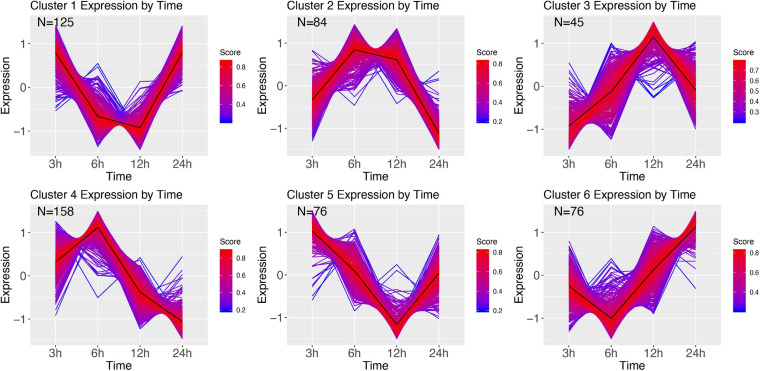
DEG clusters during *E. coli* infection over 24 h. Color indicates cluster membership score, ranging from 0 (blue) to 1 (red). Numbers in each graph represent the number of genes in this cluster with a membership score above 0.6.

Clustering of the *E. coli* time series resulted in 6 clusters (Figure 8). Cluster 1 shows initial decrease of transcription at 6 and 12 h, with an increase starting 24 h after infection. GSEA revealed this cluster to be related to protein alkylation and methylation (Supplementary Figure 5). Cluster 2 mirrors 1, wherein at 6 and 12 h after infection the genes are highly upregulated. The genes in this cluster are involved with intracellular transport, negative regulation of gene expression, and metabolism (Supplementary Figure 6). Cluster 3 starts at 3 h with genes that over time are increasingly upregulated (at 12 h), and at 24 h are baseline, and contain genes involved with the regulation of cell cycle and aromatic compound biosynthesis (Supplementary Figure 7). Cluster 4 has highest expression at 6 h, after which it decreases largely, and contains ion transmembrane transport genes, genes involved with the regulation of biological process, cellular process (Supplementary Figure 8). Cluster 5 starts with expressed genes, after which these decrease until at 12 h, and recover to the 3 h expression level at 24 h after infection. The significant GO terms associated with this cluster identified the terms: RNA modification, protein folding, biogenesis (Supplementary Figure 9). Finally, cluster 6 goes from baseline (3 h), to lower expression (6 h), and increasingly expressed for the remaining two sampling points, and contains genes involved with carbohydrate metabolism, and DNA topological change (Supplementary Figure 10).

**FIGURE 9:**
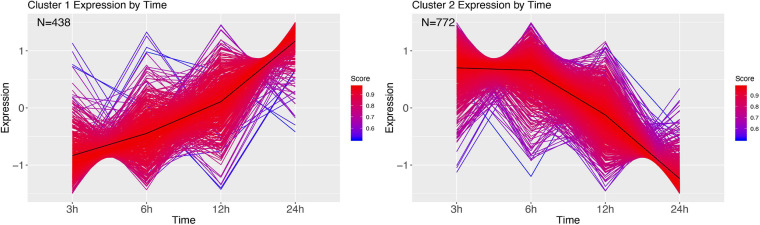
DEG clusters during *M. luteus* infection over 24 h. Color indicates cluster membership score, ranging from 0 (blue) to 1 (red).

Infection with *M. luteus* resulted in only two expression clusters over time (Figure 9). Cluster 1 show a large number of transcripts continuously increasing during the course of infection. GSEA revealed that genes involved in the defense response and aminoglycan catabolism (Supplementary Figure 11). Cluster 2 mirrors cluster 1, showing a large cluster of genes that are going down over time, and contains genes enriched for nucleoside monophosphate metabolism, metabolism and hydrogen transport (Supplementary Figure 12).

### The Immune Response

To further investigate the expression dynamics related to the immune response over 24 h after treatment, we performed a time series expression cluster analysis by the bacterial treatment. Importantly, for this analysis we removed the DE genes identified in the PBS time series when showing expression differences in the same direction (logFC > 2 or logFC < -2; FDR < 0.001; FC, fold change; FDR, false discovery rate), to allow us to more directly assess the effects of infection itself. Additionally, to identify the function of the genes identified within clusters that were not annotated in our genome, all DE transcripts were searched against the uniprot protein database.

**FIGURE 10:**
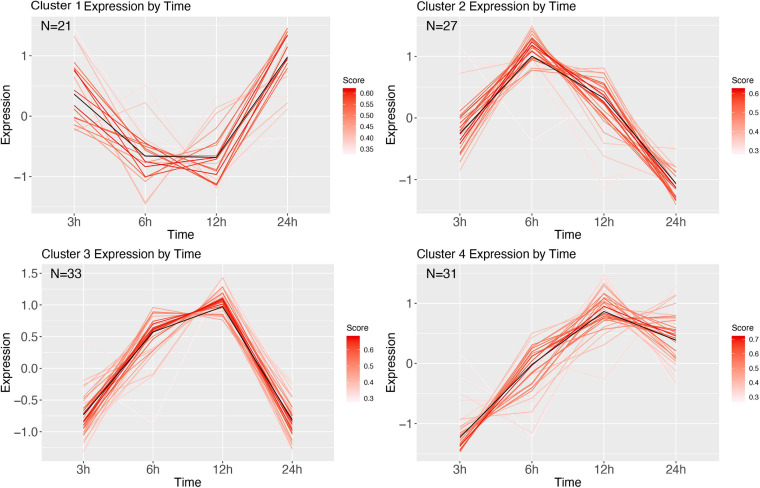
Cluster analysis over time of *E. coli* corrected with PBS. Genes were highly expressed (logFC > 2 and logFC < -2; FDR < 0.001). Each line represents a gene colored by their membership score. Numbers in each graph represent the number of genes in this cluster.

The immune response followed by an *E. coli* infection was found to have four expression clusters (Figure 10), containing 112 DE genes, of which 88 were annotated using uniprot. Cluster 1 shows decrease of expression until 12 h after infection, after which it is increasing. Within cluster 1 there were no immune genes, but rather had an Allatostatin receptor gene and Cys-loop ligand-gated ion channel subunit-like protein (Supplementary Table 4). Cluster 2 shows strong increase at 6 h, and contained the immune genes Relish (an IMD pathway signaling gene) and Hinnavin (antimicrobial peptide; Supplementary Table 4). Cluster 3 is low at 3 h, but shows relative high gene expression at 6–12 h, after which it is back to being low. The genes in this cluster with a clear immune function were antimicrobial peptides (Attacin and Moricin), and peptidoglycan recognition proteins; Supplementary Table 4). Other genes were an Endonuclease-reverse transcriptase gene, a Chitin synthase gene, an Alkaline nuclease gene, and an actin binding protein (Supplementary Table 4). The final cluster shows a similar pattern, but does not return to the lower 3 h state, and appears more baseline, and this cluster contains immune recognition genes (hemolin), modulators (serpins), as well as effector genes (antimicrobial peptides like e.g., lebocins and defensin-like peptides).

**FIGURE 11:**
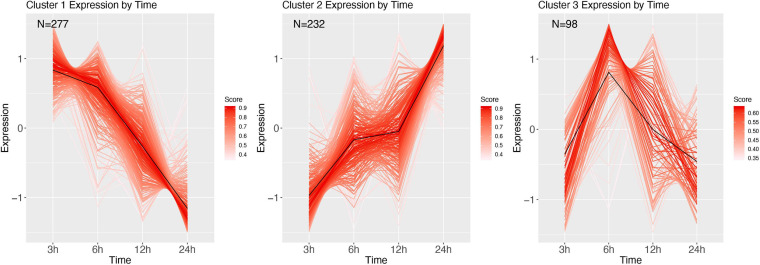
Cluster analysis over time of *M. luteus* corrected with PBS. Genes were highly expressed (logFC > 2 and logFC < -2; FDR < 0.001). Each line represents a gene colored by their membership score. Numbers in each graph represent the number of genes in this cluster.

Infection by the gram-positive *M. luteus* corrected with the PBS expression over time contained 607 DE genes, of which 463 could be annotated and divided into three clusters (Figure 11 and Supplementary Table 5). Cluster 1 reveals relatively highly expressed genes after 3 h, which over time get strongly decreased. Genes in this cluster were functionally diverse, but all appeared to be involved with homeostasis and metabolism. Cluster 2 contained genes that were increasing in expression over time and contained many immune genes. Cluster 3 starts low, after which it is strongly increasing at 6 h after infection, and returns baseline/downregulated afterward, and annotation revealed neutral lipase genes and sugar transporters.

## Discussion

Here, we found that the negative consequences of bacterial infection carry across the metamorphic boundary in the green veined white butterfly. The type of bacteria also mattered, as the detrimental effects on life history traits were stronger in *M. luteus* compared to *E. coli*. This difference between the two bacteria was already observed in the gene expression dynamics during the first 24 h after infection. Larvae infected with *M. luteus* showed a strong decrease in expression of all non-immunity related processes, with the immune system genes being strongly increased. Results of this type of “overpowering” of the organism’s homeostasis was visible also in the life history data, wherein individuals infected with *M. luteus* had the highest mortality rate, along with the lowest pupal weight, developmental rate and adult weight of all the treatments.

### Overall Survival

Mortality was a direct effect of infection. Mortality increased with an increasing dose of both pathogens and as expected, a higher dose of bacteria caused a more immediate death after infection, instead of mortality occurring at a later life-stage. Furthermore, mortality was higher with *M. luteus* than *E. coli*. These observations suggest that at lower-level infections the larvae allocate their reserves to the immune response, however, due to this reallocation, their energy reserves were significantly reduced. As a consequence of infection, mortality happening in the pupal stage suggests that these individuals died due to their inability to compensate for this loss of resources. Of the individuals that died at later stages, the majority died during eclosion, suggesting that the strain of metamorphosis was too intense, as the metamorphosis from a pupa to an adult is energetically costly. As an example, a *Manduca sexta* pupa requires 5.4 kJ of energy to complete metamorphosis, which is ∼64% of the total energy available energy in lipid stores as a final instar larva that is about to pupate (Odell, 1998). Lipid stores are also the main fuel source used during *Drosophila melanogaster* metamorphosis, using around 35% of their total lipid store just to initiate metamorphosis (Merkey et al., 2011).

Pupal diapause brings an additional energetic demand, as the preparation for diapause, and the increased lifespan due to the delayed development, depend on the energy reserves sequestered prior to the entry into diapause (Hahn and Denlinger, 2007). In *P. napi*, lipids are the main fuel source during diapause, and lipids stores show a 74% difference (in molar percentage) going from a 1-day old pupa to adult (Lehmann et al., 2016). Overall, our data showed that even if the energetic costs of an infection are met, and energetically costly metamorphosis can be completed, there are long term costs from infection, resulting in mortality due to the inability to meet the added costs of diapause.

### Hormesis at Lower Dose *E. coli* Infection

Hormesis refers to an increase in organism performance after low level exposure to agents that are commonly harmful or toxic at higher levels of exposure (Forbes, 2000). Surprisingly, during our experiment hormesis was observed, as larvae treated with a lower dose of *E. coli* showed an increased performance. Specifically, they showed the same survival rate as the control group, and had a lower mortality compared to larvae injected with PBS. Wounding causes tissue damage and the release of danger signals, and, although wounding and infection are intertwined, both show distinct signatures of gene expression (Lazzaro and Rolff, 2011; Johnston and Rolff, 2013). Our results suggest that a low-level *E. coli* infection after wounding increases survival, possibly by dual activating both wound healing and the immune response. The combination of wounding and low-level infection is likely similar to what evolutionary pressures would have responded to, since wounding and wound contamination by environmental microbes is commonplace in nature (Kamimura, 2007; Lazzaro and Rolff, 2011).

### Long Term Effects of Larval Stage Infection

A slower developmental rate as a result of infection has been well documented in previous studies on insects. Interestingly, for *P. napi*, the effects of infection on the developmental rate appeared to be dependent on the bacterial type and dose. For *E. coli*, the developmental rate was significantly longer only at the highest dose, suggesting that for these gram-negative bacteria, the larvae can compensate for lower level infections and still prioritize development. However, after infection with *M. luteus* this compensation is not performed, and subsequently their developmental rate was lower regardless of bacterial dose. *E. coli* occurs in diverse forms in nature, ranging from commensal strains to those pathogenic on human or animal hosts (van Elsas et al., 2011), and further research could identify whether perhaps these bacteria, or a bacterium closely related, is common to our butterfly. Additionally, it would be interesting to study the effects of other, more ecologically relevant gram-negative and gram-positive bacteria, to see if this difference in consequences of infection between *M. luteus* and *E. coli* is a more general pattern, wherein *P. napi* perhaps have higher tolerance to gram-negative bacteria.

After metamorphosis, several negative effects of infections remained measurable. There was a significant effect of treatment on the weight of the pupae, as well as the adult butterflies. The highest dose of *E. coli*, and all dose of *M. luteus*, had significantly lower weight than the uninjected controls. However, most of the bacterial treated individuals were not significantly lower in weight compared to the PBS injected individuals. Only males that had received the highest dose of *M. luteus* had a significantly lower weight than PBS injected males. This suggests that the injury of the injection itself does not have a lasting effect, however, the combination of injury and highest dose of bacterial treatment does. A classic tradeoff during a female adult butterfly life exists between flight performance and reproduction, and as a result the largest portion of an adult butterfly consists of reproductive reserves, stored in the abdomen, and flight muscle in the thorax (Boggs, 1981; Wickman and Karlsson, 1989; Karlsson, 1994). The abdomen of an female adult butterfly consists mostly of the reproductive organs, fat body and hemolymph, and an increase of these reserves are paralleled by a similar increase in reproductive effort for females, i.e., a larger abdomen has higher reproductive success (Wickman and Karlsson, 1989). Many studies have found trade-offs between reproduction and the immune system among insects (Boots and Begon, 1993; Thomas and Rudolf, 2010; Kingsolver and Diamond, 2011). In addition to the trade-off in females, males face a similar trade-off, with thorax weight showed sensitivity to the infection treatments, potentially negatively influencing their flight capacity and/or mating resources. In sum, our data showed that infected individuals of both sexes had smaller abdomens and thoraxes. Overall, we find that the cost of infection and wounding in the final larval instar carries over the metamorphic boundary, with adults being smaller, and most likely this would affect both their flight performance as well as their reproductive output.

### Expression Dynamics Over Time

For the gene expression analysis, larvae were injected with either PBS, or the highest dose (10^6^) of either *E. coli o*r *M. luteus*, after which sampling took place at 3, 6, 12, or 24 h after treatment. When looking across the different gene expression profiles over time, only the larva infected with *M. luteus* show a strong signal of reallocating resources to the immune system, with a strong increase of expression of genes involved with the immune system and a strong decrease of genes involved with metabolism and organismal homeostasis. These transcriptome level observations reflect the phenotypic data, which showed a higher mortality and stronger long-term effects after exposure to *M. luteus*. Additionally, the sample relationships (Figure 5) of *M. luteus* reveals that at 12 h after infection two individuals diverged from the others, most likely these individuals were on a trajectory to death.

The profiles of the larvae challenged by PBS, and larvae infected with *E. coli* showed multiple dynamic patterns, as observed in the range of expression profiles identified by the cluster analysis, had more overlapping DE genes (Figure 6) and the relationship between the samples appeared to be more similar to each other than to *M. luteus*. Wounding and pathogen infection both activate the immune system of a host, but do so by different elicitors. A sterile wound generates exclusively danger signals, which then start the immune response. Danger signals are also present when a host is challenged by a pathogen, however, they also elicit microbe-associated molecular patterns signals (MAMPs; Lazzaro and Rolff, 2011). One possible explanation for the similarity between PBS and *E. coli* expression dynamics could be that wounding is never fully sterile, and therefore the PBS treated animals could have had some low-level infection due to this treatment, and therefore, have some MAMPs signals activating a low-level immune response. It could also be that for *P. napi* the immune challenge of *E. coli* elicits a lower immune response compared to *M. luteus.*

### Metabolism and Immunity

The transcriptome of both *E. coli* and PBS showed multiple metabolic processes being up- and downregulated. However, the metabolic processes identified are known to be involved with several traits, making it challenging to interpret their role in the immune response of *P. napi.* Two type of metabolism we identified are potential interesting candidates for their involvement in the immunometabolism. First, during *E. coli* infection, ATP synthesis and carbohydrate metabolism both showed strong upregulation over time. Mounting an immune response is an energy-consuming process, and immune challenged individuals undergo a metabolic switch to enable the rapid production of ATP and new biomolecules via glucose and carbohydrate metabolism (Bajgar et al., 2015; Yang and Hultmark, 2017; Dolezal et al., 2019). Secondly, in the PBS treated animals, purine containing compound metabolism showed a strong decrease in expression. High levels of purine and pyrimidine metabolites are found during the prepupal period of *Drosophila* (An et al., 2017). Perhaps this decrease in purine metabolic expression is a switch to reallocate energy previously allotted to prepupation to wound healing. However, further research is needed to confirm this hypothesis. Overall, a multitude of metabolic processes appear to be switched on and off after infection, and provide interesting candidates for future research into the immunometabolism of *P. napi.*

### The Immune Response

To further investigate the expression dynamics related to the immune response over 24 h after treatment, we did a time series expression cluster analysis for each bacterial treatment, looking at the DE genes at that time point between the bacterial treatment, while accounting for wounding (via comparisons to the PBS treatment). This allowed us to investigate the physiological response uniquely attributed to bacterial exposure. The DE genes identified could be divided into the four general broad functional categories: pathogen-recognition genes (e.g., PGRPs), modulators (e.g., serpins and serine proteases), the genes of the signal transduction pathways (Toll, IMD), and effector genes encoding products that directly interact with microbes (e.g., antimicrobial peptides AMPs), or defense enzymes (pro-phenoloxidases). Activation of the insect immune system begins with the recognition of non-self through the activation of pattern recognition receptors (PRRs), encoded by recognition genes. After recognition of a pathogen, a sequence of modulation and signaling events is initiated. The Toll (Gram-positive) and IMD (Gram-negative) pathways are directly involved with the production of AMPs (Lemaitre and Hoffmann, 2007).

When comparing the genes involved with immune response against *E. coli* to those of *M. luteus*, several similarities were identified (compare Supplementary Table 4 with Supplementary Table 5). Hemolin, a recognition gene involved with cellular immune responses, was upregulated regardless of bacterial type. Furthermore, both bacterial treatments show upregulation of x-tox proteins. X-tox genes encode immune-related proteins with imperfectly conserved tandem repeats of defensin-like motifs. In moths, however, they have lost their antimicrobial activity, suggesting they may have some other, yet unknown, function within the systemic immune response (Girard et al., 2008; Destoumieux-Garzón et al., 2009; Mikonranta et al., 2017). Additionally, both bacterial treatments upregulated several different types of antimicrobial peptides (AMPs), suggesting that to fight off the bacteria the larvae deploys a cocktail of different AMPs, that might functionally interact and synergistically attack the bacteria. As interactions between AMP’s can be achieved either via synergism, potentiation (one AMP enabling or enhancing the activity of others), or functional diversification, i.e., combinatorial activity increasing the spectrum of responses and thus the specificity of the innate immune response (Rahnamaeian et al., 2015). Both infections resulted in upregulation of the AMPs Lebocin, Moricin, and Attacin. While both treatments showed an upregulation in common AMPs, there are a number of AMPs with treatment-specific expression profiles, produced after activation of either the Toll pathway (gram-positive bacteria) or IMD pathway (gram-negative bacteria). Heliomycin (a defensin) and lysozymes are only upregulated after *M. luteus* infection. The *E. coli* treatment showed upregulation of Hinnavin, a cecropin, which were previously found to be effective against *E. coli* (Hultmark et al., 1980). Additional variation between the expression profiles of the AMPs was found between the bacterial treatments. In the *M. luteus* treatment, all effector proteins are still strongly upregulated after 24 h, whereas for *E. coli* the expression pattern differed between the AMPs. Hinnavin, and Attacin were strongly upregulated 6–12 h after infection, whereas Lebocin was upregulated 12–24 h after infection, perhaps indicative of these AMPs potentiation.

Despite having overall similarities in the genes used during the immune response, the treatments involving the two types of bacteria also showed significantly different overall expression patterns unique to a particular type. First, phenoloxidase genes were only upregulated during the *M. luteus* infection. Secondly, the immune response against *E. coli* could be divided into four expression clusters, which showed a certain level of dynamism (Figure 10), *M. luteus* only had three clusters, which broadly could be divided into genes either being strongly downregulated (metabolic, non-immunity genes), or strongly being upregulated (immunity genes) over the 24 h (Figure 11). Interestingly, in addition to the immune genes, several other genes not directly linked to the immune system were strongly upregulated for *M. luteus*. For example, small heat shock proteins, sugar transporter proteins, cuticle proteins, and UDP-glucosyltransferase proteins showed increased expression over time. This appears to be in line with the activation of cellular immunity, which is dependent on a massive supply of glucose and glutamine (Dolezal et al., 2019). The first time-series analysis clearly revealed metabolic processes being up and down regulated for the PBS and *E. coli* treatments, however, none of these metabolic processes were unique to *E. coli*. This could imply that the effects of PBS and *E. coli* affect similar genes or allocation patterns, although it is still possible that the intensity of gene expression differs between the two treatments. In contrast, when comparing gene expression of PBS to *M. luteus*, metabolic genes showed an expression profile unique to *M. luteus*.

In sum, we found both long term and short-term effects of infection. Infection increased mortality, as well as multiple fitness parameters in individuals that survived the treatments. Furthermore, transcriptomic analysis revealed that larva infected by *M. luteus* activated several arms of the immune response, which could explain the difference in effects seen later on in the larval and adult stages. In addition, various metabolic processes were up and downregulated after both wounding and infection, providing interesting candidates for future studies looking into the immunometabolism and costs of the immune response.

## Data Availability Statement

The datasets presented in this study can be found in online repositories. The names of the repository/repositories and accession number(s) can be found below: National Center for Biotechnology Information (NCBI) BioProject, https://www.ncbi.nlm.nih.gov/bioproject/, PRJNA685195.

## Author Contributions

NK, UT, and CW conceived and designed the study, with help from LK. SN provided the additional input. NK performed the experimental work with help from LK and wrote the manuscript, with input from CW and UT. All authors read and commented on the manuscript.

## Conflict of Interest

The authors declare that the research was conducted in the absence of any commercial or financial relationships that could be construed as a potential conflict of interest.

## Footnotes

1 https://sourceforge.net/projects/bbmap
