# Supplementary material for: Physiological Tradeoffs of Immune Response Differs by Infection Type in Pieris napi
Source: Front Physiol. 2021 Jan 13;11:576797. doi: 10.3389/fphys.2020.576797 (PMC7838647; doi:10.3389/fphys.2020.576797)
Supplement: Supplementary file 1 [file Table_1.docx]

# Supplemental materials

Table of Contents

[Supplemental materials 1](#_Toc23332001)

[Supplementary Tables 1](#_Toc23332002)

[Supplementary Figures 11](#_Toc23332003)

## Supplementary Tables

SM Table 1 Number of significantly differential expressed genes between the two treatments at a false discovery rate < 0.001, at a log folc change of 2 and 0.

| **Comparison** | | | **Two LFC** | **0 LFC** |
| --- | --- | --- | --- | --- |
| E03 | vs. | PBS3 | 2 | 21 |
| E06 | vs. | PBS6 | 10 | 27 |
| E12 | vs. | PBS12 | 133 | 328 |
| E24 | vs. | PBS24 | 16 | 59 |
| M03 | vs. | PBS3 | 131 | 452 |
| M06 | vs. | PBS6 | 32 | 96 |
| M12 | vs. | PBS12 | 148 | 204 |
| M24 | vs. | PBS24 | 493 | 1336 |
|  |  |  |  |  |


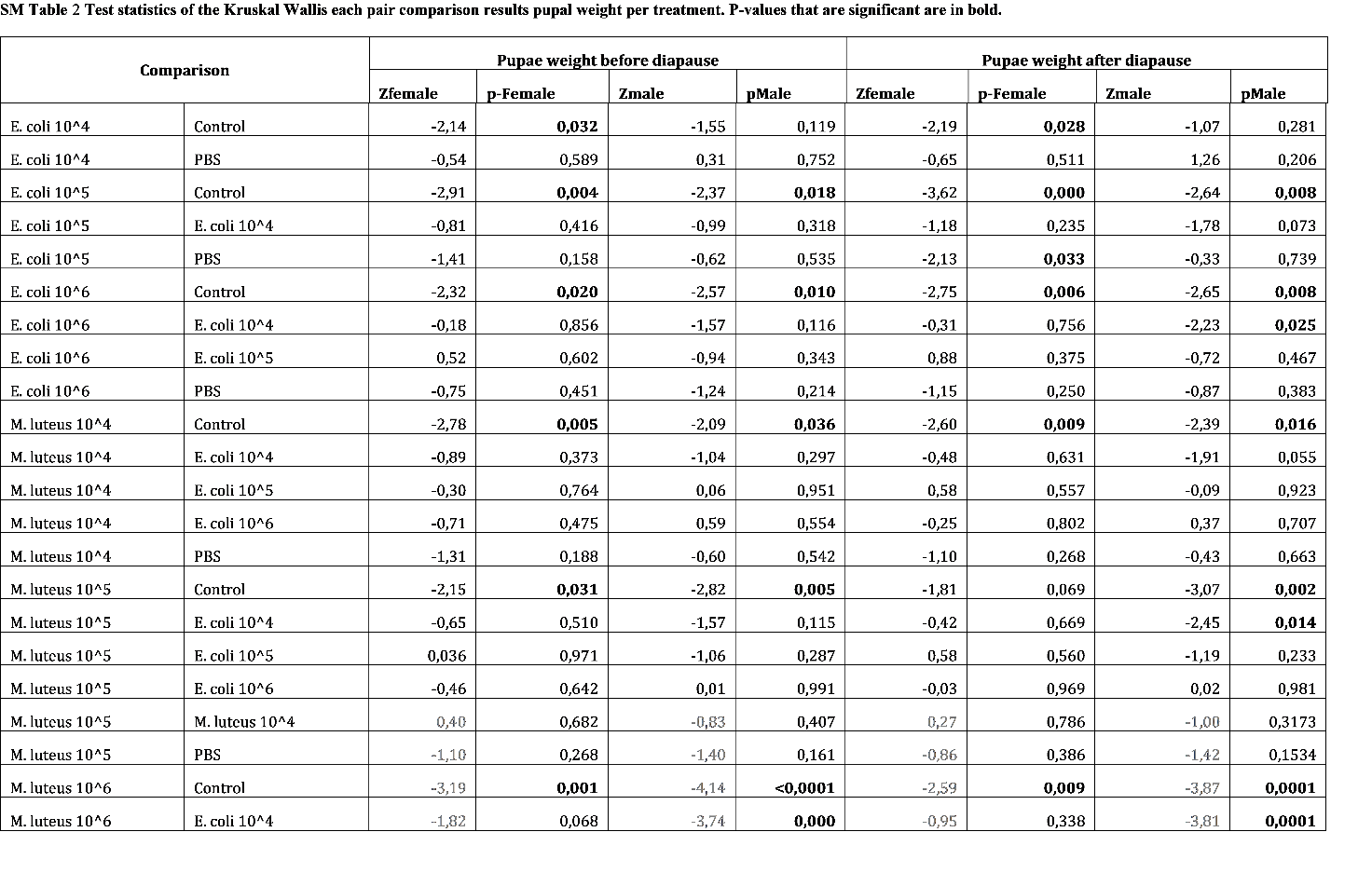


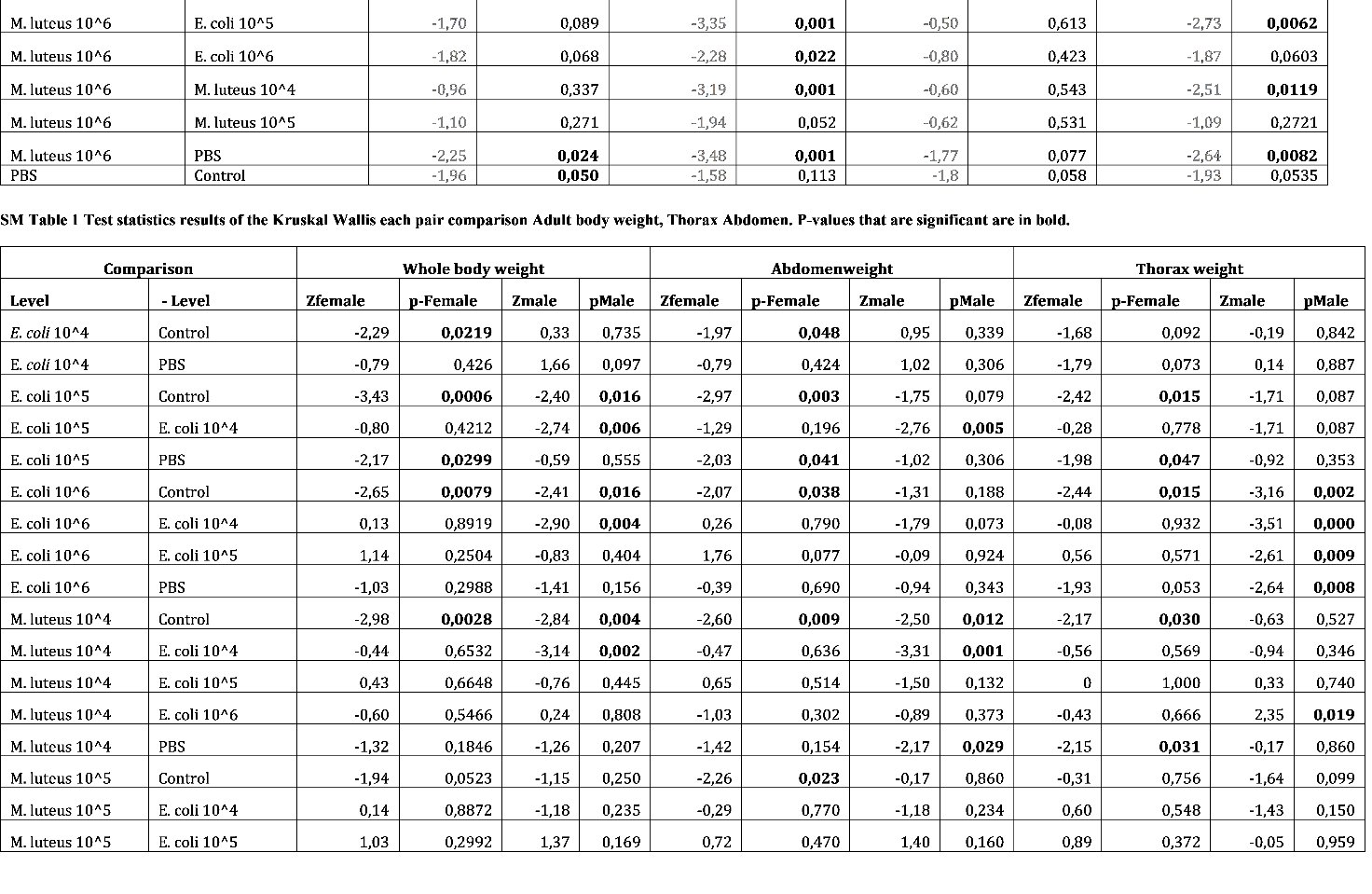


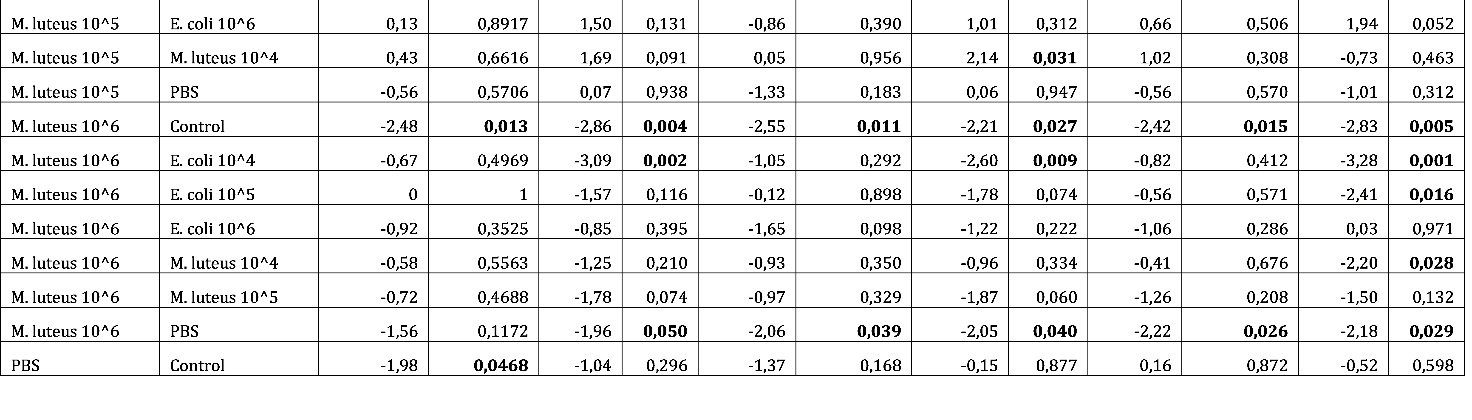


SM Table 4 Results of the gene annotations done on the genes DE in *E. coli* corrected with PBS

| gene_id | cluster | score | uniprot_name | E03 | E06 | E12 | E24 |
| --- | --- | --- | --- | --- | --- | --- | --- |
| MSTRG.24571 | 1 | 0,68 | Attacin-like antimicrobial protein | -0,85 | 0,61 | 1,09 | -0,85 |
| MSTRG.18059 | 1 | 0,66 | Endonuclease-reverse transcriptase | -0,83 | 0,70 | 1,01 | -0,89 |
| MSTRG.21526 | 1 | 0,64 | Uncharacterized protein | -0,71 | 0,58 | 1,10 | -0,97 |
| MSTRG.11624 | 1 | 0,61 | Chitin synthase | -0,90 | 0,74 | 0,98 | -0,82 |
| MSTRG.15797 | 1 | 0,60 | Antimicrobial peptide moricin | -0,94 | 0,57 | 1,11 | -0,74 |
| MSTRG.13384 | 1 | 0,59 | Alkaline nuclease | -0,63 | 0,59 | 1,09 | -1,04 |
| MSTRG.24554 | 1 | 0,57 | Attacin-like protein | -0,90 | 0,46 | 1,19 | -0,75 |
| MSTRG.3166 | 1 | 0,51 | Peptidoglycan recognition B | -1,06 | 0,62 | 1,06 | -0,62 |
| MSTRG.7993 | 1 | 0,50 | Uncharacterized protein | -0,89 | 0,89 | 0,84 | -0,84 |
| MSTRG.23753 | 1 | 0,50 | Gelsolin | -0,63 | 0,28 | 1,29 | -0,94 |
| MSTRG.18062 | 1 | 0,50 | Uncharacterized protein | -0,73 | 0,87 | 0,85 | -0,99 |
| MSTRG.3171 | 1 | 0,50 | Peptidoglycan recognition-D | -0,78 | 0,89 | 0,84 | -0,95 |
| MSTRG.10762 | 2 | 0,62 | Uncharacterized protein | -0,42 | 1,18 | 0,37 | -1,13 |
| MSTRG.22956 | 2 | 0,57 | Relish | -0,08 | 1,00 | 0,43 | -1,34 |
| MSTRG.16780 | 2 | 0,56 | BmRelish1 | 0,01 | 1,08 | 0,25 | -1,33 |
| MSTRG.12774 | 2 | 0,56 | Uncharacterized protein | 0,01 | 1,09 | 0,23 | -1,33 |
| MSTRG.15731 | 2 | 0,56 | Putative organic cation transporter | -0,16 | 1,28 | 0,04 | -1,16 |
| MSTRG.9150 | 2 | 0,55 | Uncharacterized protein | -0,24 | 0,97 | 0,56 | -1,29 |
| MSTRG.15732 | 2 | 0,55 | Putative organic cation transporter | -0,21 | 1,30 | 0,04 | -1,13 |
| MSTRG.4429 | 2 | 0,54 | Hinnavin II | -0,15 | 0,95 | 0,54 | -1,34 |
| MSTRG.11726 | 2 | 0,53 | Uncharacterized protein | 0,04 | 1,18 | 0,06 | -1,27 |
| MSTRG.12176 | 2 | 0,51 | Putative dipeptidyl-peptidase | -0,56 | 1,14 | 0,50 | -1,07 |
| MSTRG.10823 | 2 | 0,50 | Adenylate cyclase type 2 | -0,58 | 1,31 | 0,23 | -0,95 |
| MSTRG.13400 | 3 | 0,71 | Serine protease | -1,37 | -0,03 | 0,95 | 0,46 |
| MSTRG.13466 | 3 | 0,64 | Hemolin | -1,43 | 0,12 | 0,83 | 0,48 |
| MSTRG.3047 | 3 | 0,62 | Vanin-like protein 1 | -1,44 | 0,12 | 0,79 | 0,54 |
| MSTRG.23179 | 3 | 0,61 | Hemolin | -1,45 | 0,20 | 0,83 | 0,42 |
| MSTRG.3824 | 3 | 0,60 | Lebocin-like protein | -1,45 | 0,12 | 0,76 | 0,56 |
| MSTRG.12233 | 3 | 0,58 | Spod-11-tox b protein | -1,46 | 0,25 | 0,81 | 0,39 |
| MSTRG.3823 | 3 | 0,55 | Lebocin-like protein | -1,36 | -0,15 | 0,71 | 0,79 |
| MSTRG.4691 | 3 | 0,54 | Protease inhibitor-like protein | -1,19 | -0,33 | 1,17 | 0,35 |
| MSTRG.18333 | 3 | 0,54 | Serine protease | -1,20 | -0,41 | 1,02 | 0,59 |
| MSTRG.12231 | 3 | 0,54 | Antimicrobial protein 6Tox | -1,47 | 0,31 | 0,74 | 0,42 |
| MSTRG.24568 | 3 | 0,52 | Serpin-5 | -1,15 | -0,45 | 1,10 | 0,51 |
| MSTRG.7207 | 3 | 0,52 | Uncharacterized protein | -1,28 | 0,04 | 1,16 | 0,08 |
| MSTRG.3530 | 3 | 0,51 | Uncharacterized protein | -1,46 | 0,17 | 0,57 | 0,72 |
| MSTRG.14462 | 4 | 0,61 | Uncharacterized protein | 0,18 | -0,84 | -0,68 | 1,34 |
| MSTRG.4546 | 4 | 0,61 | Uncharacterized protein | 0,75 | -0,75 | -0,97 | 0,97 |
| MSTRG.20180 | 4 | 0,58 | Cys-loop ligand-gated ion channel subunit-like protein | 0,43 | -0,44 | -1,14 | 1,15 |
| MSTRG.16750 | 4 | 0,55 | Uncharacterized protein | 0,80 | -0,54 | -1,14 | 0,88 |
| MSTRG.7154 | 4 | 0,55 | Uncharacterized protein | 0,11 | -1,01 | -0,43 | 1,33 |
| MSTRG.14987 | 4 | 0,53 | Uncharacterized protein | -0,03 | -0,46 | -0,91 | 1,40 |
| MSTRG.8160 | 4 | 0,53 | Putative reverse transcriptase | 0,89 | -0,57 | -1,12 | 0,79 |
| MSTRG.4105 | 4 | 0,51 | Allatostatin receptor | -0,14 | -0,66 | -0,66 | 1,46 |

SM Table 5 Results of the gene annotations done on the genes DE in *M. luteus* corrected with PBS

| **gene_id** | **cluster** | **score** | **uniprot_name** | **M03** | **M06** | **M12** | **M24** |
| --- | --- | --- | --- | --- | --- | --- | --- |
| MSTRG.13255 | 1 | 0,90 | Storage protein 1 (Fragment) | 0,96 | 0,59 | -0,26 | -1,29 |
| MSTRG.20086 | 1 | 0,90 | Phosphoserine aminotransferase | 0,97 | 0,61 | -0,34 | -1,25 |
| MSTRG.8397 | 1 | 0,90 | Cysteine synthase | 0,99 | 0,59 | -0,32 | -1,25 |
| MSTRG.16973 | 1 | 0,90 | Sorbitol dehydrogenase | 0,89 | 0,65 | -0,22 | -1,32 |
| MSTRG.20727 | 1 | 0,89 | Putative SV2-like protein 1 | 0,93 | 0,69 | -0,39 | -1,23 |
| MSTRG.7702 | 1 | 0,88 | 2-oxoglutarate dehydrogenase | 0,92 | 0,59 | -0,17 | -1,33 |
| MSTRG.8504 | 1 | 0,88 | Sodium-dependent phosphate transporter | 0,93 | 0,69 | -0,42 | -1,21 |
| MSTRG.12057 | 1 | 0,87 | Myostatin | 0,98 | 0,52 | -0,18 | -1,32 |
| MSTRG.4804 | 1 | 0,86 | Juvenile hormone esterase | 0,80 | 0,76 | -0,25 | -1,31 |
| MSTRG.1123 | 1 | 0,86 | Acyl-coa dehydrogenase | 0,93 | 0,55 | -0,13 | -1,35 |
| MSTRG.22956 | 1 | 0,86 | Nuclear factor NF-kappa-B p110 subunit | 0,82 | 0,79 | -0,35 | -1,26 |
| MSTRG.19231 | 1 | 0,85 | Phosphoglycerate kinase | 0,85 | 0,63 | -0,12 | -1,36 |
| MSTRG.17761 | 1 | 0,85 | GMP reductase | 1,07 | 0,47 | -0,31 | -1,24 |
| MSTRG.19119 | 1 | 0,85 | S-formylglutathione hydrolase | 0,96 | 0,50 | -0,12 | -1,34 |
| MSTRG.8857 | 1 | 0,85 | Antennal esterase CXE13 | 1,07 | 0,45 | -0,25 | -1,26 |
| MSTRG.21148 | 1 | 0,84 | Alcohol dehydrogenase | 1,10 | 0,45 | -0,34 | -1,21 |
| MSTRG.21152 | 1 | 0,83 | Photoreceptor dehydrogenase | 1,10 | 0,43 | -0,30 | -1,23 |
| MSTRG.8664 | 1 | 0,83 | AAEL013642-PA | 1,02 | 0,63 | -0,51 | -1,14 |
| MSTRG.25228 | 1 | 0,83 | Citrate synthase | 1,00 | 0,65 | -0,52 | -1,14 |
| MSTRG.8264 | 1 | 0,82 | Putative uncharacterized protein | 1,01 | 0,43 | -0,10 | -1,34 |
| MSTRG.1282 | 1 | 0,82 | Regucalcin | 0,81 | 0,64 | -0,06 | -1,39 |
| MSTRG.17770 | 1 | 0,82 | Glucose-6-phosphate isomerase | 0,83 | 0,61 | -0,05 | -1,39 |
| MSTRG.15184 | 1 | 0,82 | Putative uncharacterized protein | 1,11 | 0,49 | -0,46 | -1,14 |
| MSTRG.1498 | 1 | 0,81 | Neither inactivation nor afterpotential B | 1,14 | 0,42 | -0,38 | -1,18 |
| MSTRG.9500 | 1 | 0,81 | Juvenile hormone acid methyltransferase | 0,73 | 0,77 | -0,14 | -1,36 |
| MSTRG.16556 | 1 | 0,81 | Antennal esterase CXE9 | 1,15 | 0,41 | -0,39 | -1,17 |
| MSTRG.25263 | 1 | 0,80 | Citrate synthase | 1,15 | 0,43 | -0,43 | -1,15 |
| MSTRG.13740 | 1 | 0,79 | Aldo-keto reductase | 1,07 | 0,34 | -0,11 | -1,31 |
| MSTRG.19007 | 1 | 0,79 | Drongo protein isoform 2 | 0,70 | 0,88 | -0,30 | -1,28 |
| MSTRG.13246 | 1 | 0,78 | Moderately methionine rich storage protein | 0,80 | 0,60 | 0,02 | -1,42 |
| MSTRG.21721 | 1 | 0,78 | Putative nadp transhydrogenase | 0,83 | 0,55 | 0,04 | -1,42 |
| MSTRG.21720 | 1 | 0,78 | Putative nadp transhydrogenase | 0,75 | 0,66 | 0,00 | -1,41 |
| MSTRG.23484 | 1 | 0,77 | Putative igf2 mRNA binding protein | 1,19 | 0,36 | -0,44 | -1,12 |
| MSTRG.7148 | 1 | 0,77 | Putative secreted peptide 30 | 0,67 | 0,90 | -0,29 | -1,28 |
| MSTRG.16977 | 1 | 0,77 | Sorbitol dehydrogenase | 0,85 | 0,84 | -0,58 | -1,11 |
| MSTRG.9105 | 1 | 0,76 | Putative lachesin | 0,79 | 0,58 | 0,06 | -1,43 |
| MSTRG.12176 | 1 | 0,76 | Putative dipeptidyl-peptidase | 0,66 | 0,83 | -0,12 | -1,37 |
| MSTRG.5413 | 1 | 0,76 | Uricase | 1,21 | 0,28 | -0,33 | -1,16 |
| MSTRG.8211 | 1 | 0,76 | Putative fatty acid synthase | 0,80 | 0,89 | -0,55 | -1,13 |
| MSTRG.24551 | 1 | 0,76 | Putative sugar transporter | 1,16 | 0,48 | -0,59 | -1,04 |
| MSTRG.21714 | 1 | 0,75 | Putative nadp transhydrogenase | 0,89 | 0,44 | 0,09 | -1,42 |
| MSTRG.12164 | 1 | 0,75 | Putative Cyclic AMP-dependent transcription factor ATF-6 beta | 1,23 | 0,25 | -0,33 | -1,15 |
| MSTRG.11019 | 1 | 0,75 | Putative synaptic vesicle protein | 1,13 | 0,23 | -0,07 | -1,29 |
| MSTRG.8073 | 1 | 0,74 | Enoyl-CoA hydratase | 0,72 | 0,65 | 0,07 | -1,44 |
| MSTRG.20113 | 1 | 0,73 | Dipeptidyl-peptidase | 1,01 | 0,69 | -0,71 | -1,00 |
| MSTRG.15283 | 1 | 0,72 | Myo-inositol oxygenase | 1,10 | 0,58 | -0,71 | -0,98 |
| MSTRG.17341 | 1 | 0,72 | Mitochondrial aldehyde dehydrogenase | 0,90 | 0,37 | 0,14 | -1,42 |
| MSTRG.1972 | 1 | 0,72 | Alcohol dehydrogenase | 0,63 | 0,77 | 0,02 | -1,42 |
| MSTRG.15314 | 1 | 0,71 | Fructose-bisphosphate aldolase | 0,74 | 0,55 | 0,17 | -1,46 |
| MSTRG.5989 | 1 | 0,71 | Similar to CG9701-PA | 0,64 | 0,71 | 0,09 | -1,44 |
| MSTRG.17448 | 1 | 0,70 | Triosephosphate isomerase | 0,73 | 0,54 | 0,18 | -1,46 |
| MSTRG.23357 | 1 | 0,70 | Mitochondrial aldehyde dehydrogenase | 0,63 | 0,71 | 0,11 | -1,45 |
| MSTRG.21435 | 1 | 0,70 | Putative alcohol dehydrogenase | 0,65 | 0,67 | 0,13 | -1,45 |
| MSTRG.17349 | 1 | 0,70 | Mitochondrial aldehyde dehydrogenase | 0,65 | 0,67 | 0,13 | -1,45 |
| MSTRG.24584 | 1 | 0,70 | Putative sugar transporter | 1,17 | 0,49 | -0,73 | -0,93 |
| MSTRG.12014 | 1 | 0,70 | Serpin-4A | 1,05 | 0,65 | -0,78 | -0,93 |
| MSTRG.13247 | 1 | 0,69 | Moderately methionine rich storage protein | 0,71 | 0,56 | 0,20 | -1,46 |
| MSTRG.17352 | 1 | 0,69 | Aldehyde dehydrogenase (Fragment) | 0,63 | 0,68 | 0,14 | -1,45 |
| MSTRG.21355 | 1 | 0,69 | 3-hydroxyisobutyrate dehydrogenase | 0,70 | 0,58 | 0,19 | -1,46 |
| MSTRG.5990 | 1 | 0,69 | Seminal fluid protein CSSFP028 | 0,62 | 0,69 | 0,14 | -1,45 |
| MSTRG.20161 | 1 | 0,68 | Laccase 1 | 0,58 | 1,02 | -0,39 | -1,21 |
| MSTRG.9030 | 1 | 0,68 | ATP-binding cassette transporter | 0,96 | 0,76 | -0,80 | -0,93 |
| MSTRG.12427 | 1 | 0,68 | Putative sugar transporter | 0,53 | 0,90 | -0,05 | -1,38 |
| MSTRG.3035 | 1 | 0,67 | Phosphatidylethanolamine-binding protein | 0,77 | 0,43 | 0,27 | -1,47 |
| MSTRG.25711 | 1 | 0,67 | Integrin beta pat-3 | 0,91 | 0,81 | -0,80 | -0,92 |
| MSTRG.13248 | 1 | 0,67 | Moderately methionine rich storage protein | 0,89 | 0,27 | 0,27 | -1,44 |
| MSTRG.7792 | 1 | 0,66 | Endonuclease-reverse transcriptase | 1,16 | 0,51 | -0,82 | -0,85 |
| MSTRG.3034 | 1 | 0,66 | Phosphatidylethanolamine-binding protein | 0,63 | 0,63 | 0,22 | -1,47 |
| MSTRG.19202 | 1 | 0,66 | Putative argininosuccinate synthetase | 0,73 | 0,45 | 0,29 | -1,47 |
| MSTRG.21354 | 1 | 0,66 | 3-hydroxyisobutyrate dehydrogenase | 0,73 | 0,46 | 0,29 | -1,47 |
| MSTRG.10055 | 1 | 0,66 | Mo-molybdopterin cofactor sulfurase | 0,82 | 0,33 | 0,31 | -1,46 |
| MSTRG.16239 | 1 | 0,65 | Putative sugar transporter | 1,10 | 0,59 | -0,86 | -0,83 |
| MSTRG.8561 | 1 | 0,65 | Sodium-dependent phosphate transporter | 0,57 | 0,71 | 0,19 | -1,46 |
| MSTRG.13324 | 1 | 0,65 | AGAP001085-PA (Fragment) | 0,54 | 0,75 | 0,16 | -1,45 |
| MSTRG.23395 | 1 | 0,65 | CYP9G3 | 1,32 | 0,21 | -0,68 | -0,86 |
| MSTRG.21031 | 1 | 0,64 | Cellular repressor of E1A-stimulated genes | 0,50 | 0,85 | 0,08 | -1,42 |
| MSTRG.1673 | 1 | 0,64 | Follicular epithelium yolk protein subunit | 0,54 | 0,73 | 0,19 | -1,46 |
| MSTRG.22880 | 1 | 0,64 | Peritrophin type-A domain protein 3 | 1,00 | 0,72 | -0,89 | -0,83 |
| MSTRG.16240 | 1 | 0,64 | Putative sugar transporter | 1,15 | 0,53 | -0,88 | -0,80 |
| MSTRG.14709 | 1 | 0,62 | Putative lysosomal alpha-mannosidase | 0,44 | 1,02 | -0,13 | -1,33 |
| MSTRG.24743 | 1 | 0,62 | Putative Rho-associated protein kinase | 1,18 | 0,47 | -0,91 | -0,74 |
| MSTRG.25612 | 1 | 0,61 | FK506-binding protein | 0,51 | 0,70 | 0,27 | -1,48 |
| MSTRG.5445 | 1 | 0,61 | Putative venom acid phosphatase | 1,40 | 0,05 | -0,65 | -0,79 |
| MSTRG.13249 | 1 | 0,61 | Arylphorin subunit alpha | 1,41 | -0,15 | -0,32 | -0,94 |
| MSTRG.20533 | 1 | 0,61 | Putative argininosuccinate lyase | 1,42 | -0,13 | -0,41 | -0,89 |
| MSTRG.9259 | 1 | 0,60 | Putative B-cell lymphoma 3-encoded protein | 0,41 | 1,07 | -0,20 | -1,29 |
| MSTRG.8311 | 1 | 0,60 | Neuropeptide receptor A10 | 1,35 | 0,17 | -0,81 | -0,71 |
| MSTRG.12133 | 1 | 0,60 | Putative ATP-dependent RNA and DNA helicase | 1,07 | -0,09 | 0,34 | -1,32 |
| MSTRG.10384 | 2 | 0,91 | Putative topoisomerase 1-binding RING finger | -1,10 | -0,24 | 0,02 | 1,32 |
| MSTRG.12231 | 2 | 0,91 | Heli-5-tox protein | -1,05 | -0,28 | -0,02 | 1,35 |
| MSTRG.4691 | 2 | 0,91 | Protease inhibitor-like protein | -1,12 | -0,10 | -0,09 | 1,31 |
| MSTRG.19315 | 2 | 0,90 | CYP332A1 | -1,00 | -0,31 | -0,08 | 1,38 |
| MSTRG.13466 | 2 | 0,89 | Hemolin | -0,98 | -0,30 | -0,11 | 1,39 |
| MSTRG.4901 | 2 | 0,89 | VEGF27Ca | -0,99 | -0,35 | -0,03 | 1,38 |
| MSTRG.15795 | 2 | 0,88 | Moricin-like peptide C4 | -1,00 | -0,17 | -0,22 | 1,39 |
| MSTRG.24823 | 2 | 0,88 | Serine protease inhibitor 28 | -1,10 | -0,03 | -0,19 | 1,32 |
| MSTRG.23179 | 2 | 0,87 | Hemolin | -0,94 | -0,35 | -0,12 | 1,41 |
| MSTRG.24570 | 2 | 0,87 | Attacin-like antimicrobial protein | -1,08 | -0,35 | 0,13 | 1,30 |
| MSTRG.12233 | 2 | 0,87 | Antimicrobial protein 6Tox | -0,96 | -0,40 | -0,03 | 1,39 |
| MSTRG.1020 | 2 | 0,86 | Protease inhibitor 1 | -1,21 | -0,15 | 0,14 | 1,22 |
| MSTRG.10131 | 2 | 0,86 | Putative hemolymph proteinase 5 | -0,92 | -0,40 | -0,09 | 1,41 |
| MSTRG.3171 | 2 | 0,85 | Peptidoglycan recognition protein-D | -1,24 | 0,00 | 0,03 | 1,21 |
| MSTRG.24571 | 2 | 0,84 | Attacin-like antimicrobial protein | -0,91 | -0,21 | -0,31 | 1,43 |
| MSTRG.17743 | 2 | 0,84 | WAP four-disulfide core domain protein 2 | -1,26 | -0,05 | 0,12 | 1,18 |
| MSTRG.24554 | 2 | 0,84 | Attacin-like protein | -1,26 | -0,07 | 0,14 | 1,18 |
| MSTRG.6004 | 2 | 0,83 | Heat shock protein 25.4 | -0,91 | -0,17 | -0,33 | 1,42 |
| MSTRG.19735 | 2 | 0,83 | Cytochrome P450 | -1,10 | -0,39 | 0,22 | 1,26 |
| MSTRG.10902 | 2 | 0,83 | Putative cuticle protein | -1,25 | 0,06 | 0,00 | 1,19 |
| MSTRG.3249 | 2 | 0,83 | Serine protease inhibitor 27A | -1,22 | -0,22 | 0,24 | 1,19 |
| MSTRG.3823 | 2 | 0,82 | Lebocin-like protein | -1,21 | -0,24 | 0,26 | 1,19 |
| MSTRG.21819 | 2 | 0,82 | Carboxylesterase CarE-12 | -1,04 | -0,46 | 0,22 | 1,28 |
| MSTRG.10207 | 2 | 0,82 | Putative Egl nine-like protein 1 | -1,16 | -0,32 | 0,28 | 1,21 |
| MSTRG.8784 | 2 | 0,82 | Putative cuticle protein CPH43 | -0,96 | -0,52 | 0,14 | 1,34 |
| MSTRG.6262 | 2 | 0,82 | Sugar transporter | -1,13 | -0,37 | 0,28 | 1,23 |
| MSTRG.22534 | 2 | 0,81 | Small heat shock protein 27.2 | -1,08 | 0,08 | -0,32 | 1,32 |
| MSTRG.120 | 2 | 0,81 | Aldo-keto reductase | -0,93 | -0,55 | 0,14 | 1,35 |
| MSTRG.24555 | 2 | 0,81 | Attacin | -1,23 | 0,14 | -0,13 | 1,21 |
| MSTRG.6003 | 2 | 0,81 | Small heat shock protein 27.2 | -1,01 | 0,04 | -0,38 | 1,35 |
| MSTRG.14920 | 2 | 0,80 | Chemosensory protein CSP2 | -0,99 | -0,53 | 0,23 | 1,30 |
| MSTRG.24825 | 2 | 0,80 | Serine protease inhibitor 28 | -1,20 | -0,30 | 0,32 | 1,17 |
| MSTRG.3166 | 2 | 0,79 | Peptidoglycan recognition protein B | -1,04 | -0,51 | 0,29 | 1,25 |
| MSTRG.3047 | 2 | 0,79 | Vanin-like protein 1 | -1,29 | -0,12 | 0,29 | 1,12 |
| MSTRG.16505 | 2 | 0,78 | Serine protease | -1,18 | -0,36 | 0,37 | 1,16 |
| MSTRG.21817 | 2 | 0,78 | Antennal esterase CXE5 | -0,78 | -0,61 | -0,03 | 1,42 |
| MSTRG.20339 | 2 | 0,78 | Sugar transporter | -1,03 | 0,11 | -0,41 | 1,33 |
| MSTRG.10075 | 2 | 0,78 | Dynein heavy chain | -0,82 | -0,17 | -0,46 | 1,45 |
| MSTRG.11949 | 2 | 0,78 | Hemolymph proteinase 16 | -1,19 | -0,34 | 0,38 | 1,15 |
| MSTRG.3824 | 2 | 0,77 | Lebocin-like protein | -1,30 | -0,12 | 0,32 | 1,10 |
| MSTRG.7205 | 2 | 0,76 | Tetraspanin 42Ee | -1,33 | 0,12 | 0,13 | 1,09 |
| MSTRG.25490 | 2 | 0,76 | Kazal-type inhibitor | -1,30 | 0,20 | -0,03 | 1,12 |
| MSTRG.17183 | 2 | 0,76 | Chemosensory protein | -1,33 | -0,03 | 0,29 | 1,07 |
| MSTRG.19736 | 2 | 0,75 | Cytochrome CYP341A13 | -1,06 | -0,54 | 0,40 | 1,20 |
| MSTRG.121 | 2 | 0,75 | Aldo-keto reductase | -0,67 | -0,51 | -0,31 | 1,48 |
| MSTRG.9588 | 2 | 0,74 | Prophenol oxidase activating enzyme | -1,29 | 0,26 | -0,10 | 1,12 |
| MSTRG.8975 | 2 | 0,74 | Heliomycin | -0,71 | -0,73 | 0,03 | 1,40 |
| MSTRG.24425 | 2 | 0,73 | Putative alcohol dehydrogenase | -0,73 | -0,16 | -0,57 | 1,46 |
| MSTRG.22368 | 2 | 0,72 | Putative uncharacterized protein | -1,31 | -0,16 | 0,45 | 1,02 |
| MSTRG.25289 | 2 | 0,72 | Serine proteinase-like protein 1 | -1,23 | 0,33 | -0,25 | 1,15 |
| MSTRG.18744 | 2 | 0,71 | Putative cuticle protein CPH41 | -0,57 | -0,46 | -0,47 | 1,50 |
| MSTRG.25446 | 2 | 0,71 | Lysozyme II | -1,27 | -0,28 | 0,53 | 1,02 |
| MSTRG.12868 | 2 | 0,70 | Exonuclease | -1,32 | -0,17 | 0,51 | 0,98 |
| MSTRG.10823 | 2 | 0,70 | Adenylate cyclase type 2 | -0,99 | 0,26 | -0,55 | 1,28 |
| MSTRG.23724 | 2 | 0,69 | UDP-glucosyltransferase | -0,88 | -0,77 | 0,45 | 1,20 |
| MSTRG.125 | 2 | 0,69 | Aldo-keto reductase | -0,78 | -0,83 | 0,36 | 1,25 |
| MSTRG.24556 | 2 | 0,69 | Attacin-like protein | -1,29 | 0,39 | -0,17 | 1,07 |
| MSTRG.21859 | 2 | 0,68 | EP1-like protein | -1,36 | 0,35 | -0,01 | 1,01 |
| MSTRG.24552 | 2 | 0,67 | Attacin | -1,42 | 0,21 | 0,27 | 0,94 |
| MSTRG.22535 | 2 | 0,67 | Small heat shock protein 27.2 | -1,40 | 0,33 | 0,12 | 0,95 |
| MSTRG.9514 | 2 | 0,66 | EP1like2 protein | -0,54 | -0,22 | -0,71 | 1,47 |
| MSTRG.22195 | 2 | 0,65 | Putative cuticle protein CPH43 | -0,38 | -0,69 | -0,42 | 1,49 |
| MSTRG.3358 | 2 | 0,65 | UDP-glucosyltransferase | -1,01 | -0,69 | 0,67 | 1,03 |
| MSTRG.9975 | 2 | 0,64 | Alpha-esterase | -0,38 | -0,91 | -0,13 | 1,42 |
| MSTRG.21956 | 2 | 0,63 | Yellow-d | -1,10 | 0,48 | -0,51 | 1,13 |
| MSTRG.14212 | 2 | 0,62 | Serine protease inhibitor 5 | -1,15 | -0,52 | 0,77 | 0,90 |
| MSTRG.15797 | 2 | 0,62 | Antimicrobial peptide moricin | -1,20 | 0,55 | -0,39 | 1,05 |
| MSTRG.10122 | 2 | 0,61 | Putative hemolymph proteinase 5 | -0,22 | -0,85 | -0,38 | 1,45 |
| MSTRG.14358 | 3 | 0,63 | Neutral lipase | -0,71 | 1,43 | -0,05 | -0,68 |
| MSTRG.24845 | 3 | 0,62 | Zinc finger MYM-type protein 1 (Fragment) | -0,77 | 1,28 | 0,30 | -0,82 |
| MSTRG.11256 | 3 | 0,61 | Putative uncharacterized protein | -0,71 | 1,25 | 0,36 | -0,90 |
| MSTRG.15732 | 3 | 0,61 | Putative organic cation transporter | -0,77 | 1,20 | 0,44 | -0,87 |
| MSTRG.4364 | 3 | 0,61 | Ebony | -0,94 | 1,22 | 0,40 | -0,68 |
| MSTRG.6113 | 3 | 0,60 | Sugar transporter | -0,88 | 1,19 | 0,46 | -0,77 |

## Supplementary Figures

| **May 2014** | **June 2014** | **July-Dec** | **January 2015** |
| --- | --- | --- | --- |
| **Experimental treatment** | **Pupation** | **Diapause** | **Eclosion** |
| - Weight larva - Time to pupation | - Weight 23 days old |  | - Weight 247 day old pupa - Whole body mass adult - Abdomen weight - Thorax weight |

SM Figure 1 Graphical representation of the different treatments for the life history experiment

SM Figure 2 Revigo visualization of the enriched GO terms associated of PBS cluster 1

SM Figure 3 Revigo visualization of the enriched GO terms associated of PBS cluster 2

SM Figure 4 Revigo visualization of the enriched GO terms associated of PBS cluster 3

SM Figure 5 Revigo visualization of the enriched GO terms associated of *E. coli* Cluster 1

SM Figure 6 Revigo visualization of the enriched GO terms associated of *E. coli* cluster 2

SM Figure 7 Revigo visualization of the enriched GO terms associated of *E. coli* cluster 3

SM Figure 8 Revigo visualization of the enriched GO terms associated of *E. coli* cluster 4

SM Figure 9 Revigo visualization of the enriched GO terms associated of *E. coli* cluster 5

SM Figure 10 Revigo visualization of the enriched GO terms associated of *E. coli* cluster 6

SM Figure 11 Revigo visualization of the enriched GO terms associated of *M. luteus* cluster 1

SM Figure 12 Revigo visualization of the enriched GO terms associated of *M. luteus* cluster 2
